# Serum Oxytocin Levels and an Oxytocin Receptor Gene Polymorphism (rs2254298) Indicate Social Deficits in Children and Adolescents with Autism Spectrum Disorders

**DOI:** 10.3389/fnins.2017.00221

**Published:** 2017-04-21

**Authors:** Shuhan Yang, Xiaopeng Dong, Xuan Guo, Yu Han, Hanbing Song, Lei Gao, Wei Dai, Yuanyuan Su, Xin Zhang

**Affiliations:** ^1^Department of Maternal, Child and Adolescent Health, School of Public Health, Tianjin Medical UniversityTianjin, China; ^2^Department of Applied Science, The College of William and MaryWilliamsburg, VA, USA

**Keywords:** autism spectrum disorders, oxytocin, oxytocin receptor gene, rs2254298, social deficits, children and adolescents

## Abstract

The neuropeptide oxytocin (OT) and its receptor (*OXTR*) have been predicted to be involved in the regulation of social functioning in autism spectrum disorders (ASD). Objective of the study was to investigate serum OT levels and the *OXTR* rs2254298 polymorphism in Chinese Han children and adolescents with ASD as well as to identify their social deficits relevant to the oxytocinergic system. We tested serum OT levels using ELISA in 55 ASD subjects and 110 typically developing (TD) controls as well as genotyped the *OXTR* rs2254298 polymorphism using PCR-RFLP in 100 ASD subjects and 232 TD controls. Autistic symptoms were assessed by the Autism Behavior Checklist (ABC) and the Childhood Autism Rating Scale (CARS). There were no significant associations between *OXTR* rs2254298 polymorphism and ASD, serum OT levels and age, as well as serum OT levels and intelligent quotient (IQ) in both ASD and TD groups. However, ASD subjects exhibited elevated serum OT levels compared to TD controls and positive correlations between serum OT levels and “adaptation to change score” in the CARS and CARS total scores. Moreover, in the ASD group, significant relationships were revealed between the single-nucleotide polymorphism (SNP) rs2254298 and serum OT levels, the category “stereotypes and object use” in the ABC and the category “adaptation to change” in the CARS. These findings indicated that individuals with ASD may exhibit a dysregulation in OT on the basis of changes in *OXTR* gene expression as well as environmentally induced alterations of the oxytocinergic system to determine their social deficits.

## Introduction

Autism spectrum disorders (ASD) are a set of complex neurodevelopmental disorders characterized by deficits in social interaction and communication as well as restricted, repetitive behaviors (American Psychiatric Association, 2013). A recent epidemiological survey estimated the prevalence of ASD to be one in 68 children, with a male: female ratio of 4.5: 1 (CDC, 2014). The etiology of ASD likely includes both genetic heritability and environmental risk factors (Hallmayer et al., 2011; Ronald and Hoekstra, 2011), and twin studies have demonstrated an estimated heritability of 70–90% (Freitag, 2011; Robinson et al., 2012). ASD result in negative effects on physical health and quality of life among the ASD population as well as heavy burdens to their families and society.

A wealth of literature indicates intense interest in the function of oxytocin (OT) in affiliation and social behavior in both animals and humans (Carter, 1998; Donaldson and Young, 2008; Insel, 2010). Some studies have also suggested a potential association between a primary dysfunction of the central OT system and core symptoms of social deficits in ASD (Insel et al., 1999; Lukas and Neumann, 2013). Because previous work has implied the important role of oxytocin receptor (*OXTR*) in the modulation of OT activities and the OT-*OXTR* signaling may be involved in ASD pathophysiology (Yamasue, 2013), the *OXTR* gene is notable as a potential candidate gene for ASD. These findings have attracted researchers to investigate whether genetic variation in human *OXTR* gene is associated with social deficits in ASD.

OT is a nonapeptide with a highly conserved structure and is mainly synthesized in magnocellular neurons in the paraventricular and supraoptic nuclei of the mammalian hypothalamus. OT affects peripheral tissues and central brain regions, including the central *OXTR* network. Centrally projecting neuropeptidergic neurons target regions that regulate stress coping, moods, and social behaviors (Taurines et al., 2014). In addition, OT has been reported to be involved in attachment, pair bonding, sexual reproduction, maternal behavior, stress response, social memory, emotional cognition, and social interaction (Insel and Fernald, 2004; Kosfeld et al., 2005; Lee et al., 2009). Metabolic abnormalities of OT (Green et al., 2001) have exhibited a significant association with impairment in social interaction and communication in ASD individuals.

These outcomes indicate that OT may play a role in the etiology of ASD, especially in the social impairment domain. Several exogenous OT treatments have managed to normalize impaired social functioning among individuals with ASD. Adult ASD patients exhibited reduced repetitive behaviors (Hollander et al., 2003) and enhanced affective speech comprehension (Hollander et al., 2007) after intravenous OT infusion. Improved emotional recognition from eye regions in ASD boys was reported after intranasal OT administration (Guastella et al., 2010). Moreover, increased cooperation in simulated ball games, feelings of trust with teammates and time spent gazing at the eye regions of unfamiliar faces (Andari et al., 2010), as well as improvements in social cognition and quality of life (Anagnostou et al., 2012) have been reported in adult ASD patients after OT nasal sprays. These findings provide evidence that OT may have the potential to improve human social cognition and behavior and to partially restore the damaged social cognitive neurological functions (Meyer-Lindenberg et al., 2011; Striepens et al., 2011). Nevertheless, recent double-blind, randomized, placebo-controlled studies in ASD individuals did not disclose significant clinical efficacies of intranasal OT on social cognition or behavior. For example, compared to the placebo group, Dadds et al. (2014) demonstrated that the intranasal OT treatment for male youths with ASD did not bring significant improvements on social interaction skills, emotion recognition, or general behavioral adjustment. Guastella et al. (2015) also did not find the efficacy of OT nasal spray for youth with ASD on social responsiveness and social cognition. Moreover, Althaus et al. (2015) detected no significant treatment effects of OT nasal spray for male adults on empathic arousal responses to affective social pictures. Therefore, it is essential to discover the real relationship between the oxytocinergic system and ASD.

Experiments have been designed to measure plasma OT levels in individuals with ASD to compute pertinent correlations. Relative to typically developing (TD) controls, several groups have reported lower plasma OT levels in individuals with ASD (Modahl et al., 1998; Green et al., 2001; Al-Ayadhi, 2005; Andari et al., 2010). However, there are also studies demonstrating elevated plasma OT levels in ASD participants (Jansen et al., 2006; Jacobson et al., 2014). Furthermore, a third group of studies reported no differences in plasma OT levels between ASD cases and unaffected controls (Miller et al., 2013; Parker et al., 2014; Taurines et al., 2014). There still remains questions that whether low or high plasma OT levels act as a biomarker of ASD and/or social deficits.

The *OXTR* gene is located on chromosome 3p25, spans 17 kb and includes four exons and three introns (Inoue et al., 1994). It encodes a member of the class I family of G protein-coupled receptors, which are characterized by seven transmembrane domains. Oxytocin receptors are concentrated in brain regions involved in social behavior, including the olfactory bulbs, pyriform cortex, amygdala, and lateral septum (Ferguson et al., 2000). Genome-wide linkage studies (McCauley et al., 2005; Lauritsen et al., 2006) have suggested that the *OXTR* gene is a plausible candidate gene for ASD. In addition, polymorphisms in the *OXTR* gene may affect human social cognition and attachment (Chen et al., 2011; Wu et al., 2012) and abnormalities in the *OXTR* gene have been proposed to cause social disorders in individuals with ASD (Choleris et al., 2007; Lerer et al., 2008). The association between *OXTR* gene polymorphisms and the etiology of ASD has drawn massive attention. At present, a single-nucleotide polymorphism (SNP) in the third intron, rs2254298, has been identified as a particularly promising candidate to explain differences in oxytocinergic functioning (Meyer-Lindenberg et al., 2011), especially in social functioning (Costa et al., 2009; Chen et al., 2011; Thompson et al., 2011; Wu et al., 2012). Comparative genetics imply that a mutation replacing guanine (G) by adenine (A) of *OXTR* SNP rs2254298 has occurred at some point during human evolution. In European populations, the vast majority of individuals are homozygous for the G allele, but in Asian populations, the frequency of GG genotype is around just 40–50% compared with the frequencies of AA and AG genotypes (Chelala et al., 2009). Reasons for the selection of SNP rs2254298 in our study were on the basis of its special function and the inconsistent conclusions of aforetime studies involving ethnically distinct study populations: a Chinese Han population (Wu et al., 2005), a Caucasian population in the United States (Jacob et al., 2007), Swiss (Nyffeler et al., 2014), and northern European populations (Di Napoli et al., 2014), a Japanese population (Liu et al., 2010), an Israeli population (Lerer et al., 2008) and a German population (Wermter et al., 2010). For instance, studies on Chinese (Wu et al., 2005) and Japanese (Liu et al., 2010) populations discovered that the “A” allele of rs2254298 was associated with ASD. However, Jacob et al. (2007) found the “G” allele of rs2254298 as a risk allele in Caucasian ASD population. These results need further studies to clarify the role of *OXTR* rs2254298 polymorphism in ASD.

Although recent evidence supporting the essential role of OT and the *OXTR* gene in social functioning in ASD individuals has been reported, no consensus has been made on the associations among OT levels, the *OXTR* SNP rs2254298 and ASD. In order to clarify whether significant differences of serum OT levels and *OXTR* rs2254298 polymorphism would exhibit between ASD and TD groups, as well as seek out the interaction of serum OT levels and *OXTR* rs2254298 polymorphism and their impacts on social deficits of ASD subjects in Chinese Han children and adolescents, the present case-control study tested serum OT levels using ELISA, detected the presence of the *OXTR* rs2254298 polymorphism using PCR-RFLP and measured the ASD social phenotypes using the ABC and CARS scales in ASD subjects and TD controls of both genders. We analyzed the differences of serum OT levels between the ASD and TD groups, the correlations between serum OT levels and age, intelligent quotient (IQ), the ABC scores and the CARS scores. Moreover, the association between the *OXTR* rs2254298 polymorphism and ASD, as well as the relationships between *OXTR* SNP rs2254298 genotypes and serum OT levels, the ABC scores and the CARS scores were also analyzed. Finally, we hypothesized that Chinese Han children and adolescents with ASD may exhibit a dysregulation in OT on the basis of changes in *OXTR* gene expression as well as environmentally induced alterations of the oxytocinergic system to determine their social deficits.

## Materials and methods

### Participants

The study was approved by the Tianjin Medical University Institutional Review Board and written informed consent was obtained from school principals, parents and/or caregivers after a clear elaboration of detailed information. We recruited Chinese Han children and adolescents with ASD from the local special education schools and Chinese Han TD controls from the local mainstream schools in Tianjin. Serum OT levels were measured by ELISA in 55 ASD subjects (43 males and 12 females) and 110 age- and gender-matched TD controls (86 males and 24 females) between 2 and 17 years of age. The *OXTR* rs2254298 polymorphism was genotyped by PCR-RFLP in 100 ASD subjects (80 males and 20 females) and 232 age- and gender-matched TD controls (186 males and 46 females) between 2 and 18 years of age. Participants for ELISA (ASD group, *n* = 55; TD group, *n* = 110) were a subset of participants for PCR-RFLP (ASD group, *n* = 100; TD group, *n* = 232). ASD subjects were diagnosed by qualified and experienced psychologists, psychiatrists or neurologists according to the Diagnostic and Statistical Manual of Mental Disorders, Fourth Edition (DSM-IV) criteria (American Psychiatric Association, 1996) in the major hospitals of Tianjin and Beijing, which was conformed with the Childhood Autism Rating Scale (CARS) (Schopler et al., 1980) by clinical pediatric psychiatrists in Tianjin. A CARS total score of ≥30 is the cutoff for distinguishing children “at risk” for autism from pervasive developmental disorder-not otherwise specified (PDD-NOS) (Schopler et al., 1988; Chlebowski et al., 2010), and a CARS total score of ≥25.5 is indicative of ASD (Chlebowski et al., 2010). Participant characteristics are reported in Table 1. The Autism Behavior Checklist (ABC) was also administered for the assessment of their autistic symptoms (Rellini et al., 2004). Moreover, intelligent quotient (IQ) was determined using the Chinese Binet Scale (Binet) (Wu, 1982) by clinical pediatric psychologists in Tianjin. ASD subjects were included with a full-scale IQ of ≥30 and TD controls were included with an IQ in the average range. Moreover, 6 ASD subjects for ELISA and 9 ASD subjects for PCR-RFLP had an IQ of >70. In the present study, none of the ASD subjects fulfilled a DSM-IV diagnosis (American Psychiatric Association, 1996) of any mental disorders (schizophrenia, attention deficit/hyperactivity disorder, depressive disorder, and bipolar disorder), as well as suffered from epileptic seizures and fragile X syndrome on the basis of medical history reported by parents. TD controls were required to be free of psychiatric, neurological or physical disorders in the present or past on the basis of medical history reported by parents and with a score of <7 on the Clancy Autism Behavior Scale (CABS) (Clancy et al., 1969). To ensure the robustness of the experiments, none of the participants in any group were taking special drugs, such as psychotropic or immune-altering medications.

**Table 1:** **Participant characteristics**.

**Participants**	**Total (*n*)**	**Sex (*****n*****)**		**Age (year)**	**CARS total score**	**Full-scale IQ**
		**Male**	**Female**			
**ELISA FOR OT**						
ASD group	55	43	12	7.73 ± 0.56	32.56 ± 0.48	59.65 ± 1.76
Autism	45	34	11	7.96 ± 0.65	33.56 ± 0.47	56.27 ± 1.59
Severe autism	9	7	2	11.78 ± 1.33	38.78 ± 0.40	49.11 ± 3.85
Mild/moderate autism	36	27	9	7.00 ± 0.66	32.25 ± 0.30	58.06 ± 1.63
PDD-NOS	10	9	1	6.70 ± 0.99	28.10 ± 0.31	74.90 ± 3.85
TD group	110	86	24	7.75 ± 0.24		101.70 ± 0.71
**PCR-RFLP FOR** ***OXTR***						
ASD group	100	80	20	8.52 ± 0.42	33.30 ± 0.44	56.25 ± 1.49
Autism	83	65	18	8.94 ± 0.46	34.41 ± 0.44	52.25 ± 1.25
Severe autism	25	20	5	10.28 ± 0.82	39.88 ± 0.43	43.08 ± 1.83
Mild/moderate autism	58	45	13	8.36 ± 0.55	32.05 ± 0.21	56.21 ± 1.30
PDD-NOS	17	15	2	6.47 ± 0.76	27.88 ± 0.27	75.76 ± 3.61
TD group	232	186	46	8.42 ± 0.25		102.06 ± 0.50

*Participants for ELISA (ASD group, n = 55; TD group, n = 110) were a subset of participants for PCR-RFLP (ASD group, n = 100; TD group, n = 232). The values are expressed in mean ± SEM. ASD, autism spectrum disorders; CARS, Childhood Autism Rating Scale; ELISA, enzyme-linked immunosorbent assay; IQ, intelligent quotient; n, number; OT, oxytocin; OXTR, oxytocin receptor; PCR-RFLP, polymerase chain reaction-restriction fragment length polymorphism, PDD-NOS, pervasive developmental disorder-not otherwise specified, TD, typically developing*.

### Assessment of autistic symptoms

#### Autism Behavior Checklist (ABC)

The Autism Behavior Checklist (ABC) is a behavior checklist that consists of 57 items in 5 categories: “sensory”, “relating”, “stereotypes and object use”, “language”, and “social and self-help”. Each item corresponds to a single score referring to a single symptomatological area (Rellini et al., 2004). The scale utilizes an observer's rating of a series of typical autistic behaviors in a certain subject and provides advice for educational intervention.

#### Childhood Autism Rating Scale (CARS)

The Childhood Autism Rating Scale (CARS) is a 15-item behavior rating scale that includes 14 domains plus one category of general impressions that are rated on a four-point scale (1 = appropriate for age; 2 = mildly abnormal; 3 = moderately abnormal; 4 = severely abnormal) according to interaction and observation. The 15 items are as follows: “relating to people”, “imitative behavior”, “emotional response”, “body use”, “object use”, “adaptation to change”, “visual response”, “listening response”, “perceptive response”, “fear or anxiety”, “verbal communication”, “non-verbal communication”, “activity level”, “level and consistency of intellective relations”, and “general impressions” (Rellini et al., 2004). The CARS is widely used by psychiatrists to identify children with autism and as a further measurement of the severity of this disease. Total scores can range from a low of 15 to a high of 60; scores of less than 30 indicate that the individual is in the non-autistic range, scores between 30 and 36.5 indicate mild/moderate autism, and scores from 37 to 60 indicate severe autism (Schopler et al., 1988).

### Sample collection

Participants were previously informed to fast in the morning before sampling to avoid the influence of food and/or drink.

Five milliliters of venous blood were collected from each participant's antecubital region by trained and qualified nurses between 8:00 and 9:30 a.m. in pro-coagulation tubes with yellow caps (Becton, Dickinson and Company). The tubes were gently inverted five times immediately and left undisturbed at room temperature for 30 min. Then, they were kept on ice until centrifuging at 1,731 × g for 10 min at 4°C. Serum was isolated and divided into 1-ml aliquots and immediately stored at −80°C until assay.

DNA samples were obtained by an experienced technician by taking a sterile swab and rubbing it against the inside of participants' cheeks and placing it into a labeled sterile 2-ml Falcon tube.

### DNA extraction

DNA was extracted from the swabs according to the manufacturer's protocol for the Swab Gen DNA Kit (CW0530, CWBIO, Beijing, China). The DNA solution for the current experiment was then stored at 4°C, and the redundant stock was stored at −20°C. The concentration of each DNA sample was determined using a NanoDrop ND-2000 spectrophotometer (Thermo Scientific).

### Oxytocin enzyme-linked immunosorbent assay (ELISA)

Serum sample extraction procedures were previously carried out to decrease the potential cross-reacting molecules. A 1:1 mixture of serum sample and 0.1% trifluoroacetic acid in water (TFA-H_2_O) was centrifuged at 17,000 × g for 15 min at 4°C. Supernatants were applied to the Sep-Pak C18 columns (Waters Corporation Milford, Massachusetts USA) and eluted off with a solution (acetonitrile/0.1% TFA-H_2_O ratio 60:40). Samples was evaporated at 4°C and stored at −20°C until reconstitution. Serum OT levels were measured using commercially available ELISA Kits (O30152-09, Beijing Biotopped Science & Technology CO., Ltd., China), all steps in the manufacturer's instructions were followed, and results were read under 450 nm wavelength on a Power Wave-I microplate spectrophotometer (Sunrise-Basic BioTek, Tecan). All assays were performed in duplicate. Standard curves and concentrations were computed by Excel. The assay range was from 2 pg/ml to 600 pg/ml. The intra-assay coefficient of variation was 6.9%, and the inter-assay coefficient of variation was 11.8%.

### Genotyping

The *OXTR* SNP rs2254298 was amplified by using polymerase chain reaction-restriction fragment length polymorphism (PCR-RFLP) analysis. The primer sequences were as follows: 5′-TGA AAG CAG AGG TTG TGT GGA CAG G-3′ and 5′-AAC GCC CAC CCC AGT TTC TTC-3′ (Wu et al., 2005). PCR was performed on a C1000 Thermal Cycler (Bio-Rad, Hercules, CA, USA) in a 20-μl volume consisting of 0.1 μl Taq (Takara, Code. #DR001A), 2 μl 10 × PCR buffer (with Mg^2+^), 1.6 μl dNTP Mixture (2.5 mM), 1 μl each of the forward and reverse primers (10 μM/L), 3 μl genomic DNA, and 11.3 μl ddH_2_O. The PCR process included an initial denaturation step at 95°C for 5 min; 35 cycles of denaturation at 95°C for 30 s, annealing at 64.5°C for 30 s and extension at 72°C for 1 min; followed by a final extension for 7 min at 72°C. PCR products that were 307 bp long were visualized on a 2% agarose gel after electrophoresis. After the PCR products were digested at 65°C for 20 min with 0.5 μl of the restriction enzyme BsrI (New England Biolabs, Cat. #R0527), the samples were run at 80 V for 90 min on an 8% polyacrylamide gel (acrylamide/bis-acrylamide ratio 19:1) and were visualized to distinguish the G allele (bands of 164, 101, 34, and 8 bp) from the A allele (bands of 164, 135, and 8 bp). To ensure the accuracy of the RFLP profiles, gene sequencing was performed by Genewiz Inc, and the sequencing results agreed with the PCR-RFLP results.

### Statistical analysis

The results were analyzed using SPSS17.0 software (IBM Corporation New York, USA) and SAS version 9.3.2 (SAS Institute Inc, Cary, NC). Kolmogorov-Smirnov test was performed to determine the normal distribution of the data. Levene's test was utilized to determine the homogeneity of variance. Differences in serum OT levels between two groups were examined with a Mann-Whitney U test and spearman rank-order correlations (including spearman partial correlation) were used to evaluate strength of associations. The Hardy-Weinberg equilibrium for the genotype frequency distributions of rs2254298 was assessed using a χ^2^ goodness-of-fit test. A χ^2^-test was also used in analyzing the genotype and allele frequencies, with a significance level of α = 0.05. A one-way analysis of covariance (ANCOVA) with sex, age, and IQ as covariates was applied for comparisons of ABC scores among three *OXTR* SNP rs2254298 genotypes. Kruskal-Wallis *H* tests were utilized for comparisons of serum OT levels and CARS total scores among three *OXTR* SNP rs2254298 genotypes. A ordinal polytomous logistic regression analysis was performed to discover the differences of the scores of each item in the CARS among three *OXTR* SNP rs2254298 genotypes (while adjusting for sex, age, and IQ). Odds ratios (ORs) were expressed with 95% confidence intervals (95% CI). *Post-hoc* Bonferroni correction for multiple comparisons was performed. A *p* < 0.05 was considered significant.

## Results

### Serum oxytocin (OT) levels

**Figure 1:**
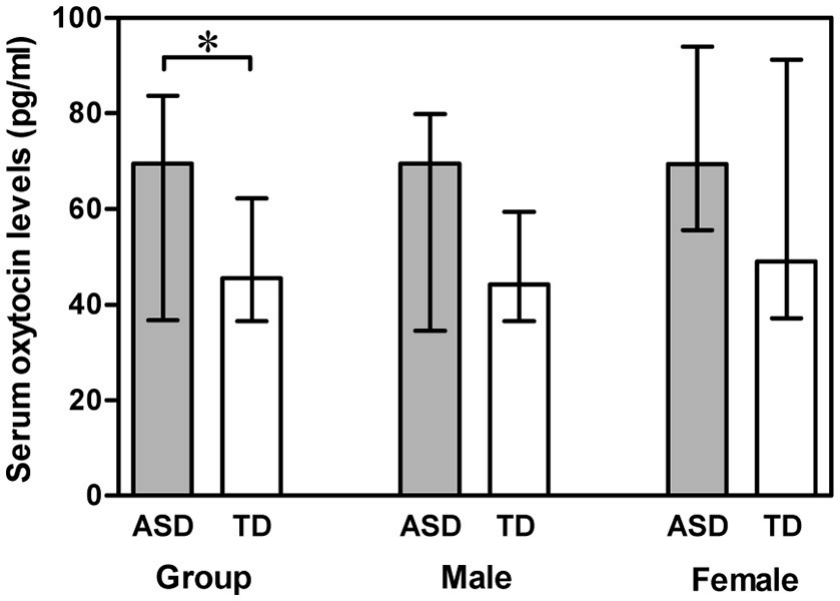
**Vertical bar charts of serum oxytocin levels across diagnosis (ASD compared with TD) and genders (males compared with females) with median (IQR)**. Error bars show interquartile range (IQR). Group: *n* = 55 ASD and *n* = 110 TD. Male: *n* = 43 ASD and *n* = 86 TD. Female: *n* = 12 ASD and *n* = 24 TD. The ASD group exhibited significantly higher serum oxytocin levels than the TD group.^*^p < 0.05. *ASD, autism spectrum disorders; IQR, interquartile range; TD, typically developing*.

A Mann-Whitney U test was used to assess the differences between ASD subjects and TD controls. Serum OT levels was significantly higher in the ASD group compared with the TD group (*Z* = −2.178, *p* = 0.029). However, gender had no significant effect on serum OT levels in any comparison, between the two groups (males *Z* = −1.694, *p* = 0.090; females *Z* = −1.309, *p* = 0.191) and between males and females within the ASD group (*Z* = −1.100, *p* = 0.271) and within the TD group (*Z* = −1.230, *p* = 0.219) (Figure 1). Correlations between serum OT levels and age, as well as IQ are presented in Table 2 with unadjusted and adjusted spearman rank-order correlations. The positive correlations between serum OT levels and age of all ASD subjects, as well as ASD males vanished after Spearman partial correlation. No associations were found between serum OT levels and IQ with both analyses in ASD and TD groups.

**Table 2:** **Correlations between serum OT levels and age, as well as IQ in ASD and TD groups**.

**Group**	**Spearman correlation**		**Spearman partial correlation**		
	**Serum OT levels**		**Confounding factors**	**Serum OT levels**	
	***r***	***p***		***r***	***p***
**ASD-AGE**					
Total (*n* = 55)	0.270	**0.046**	sex + IQ	0.202	0.147
Male (*n* = 43)	0.349	**0.022**	IQ	0.263	0.093
Female (*n* = 12)	−0.098	0.761	IQ	−0.050	0.883
**TD-AGE**					
Total (*n* = 110)	−0.085	0.377	sex + IQ	−0.079	0.417
Male (*n* = 86)	−0.086	0.430	IQ	−0.078	0.478
Female (*n* = 24)	−0.160	0.455	IQ	−0.138	0.529
**ASD-IQ**					
Total (*n* = 55)	−0.216	0.113	sex + age	−0.078	0.577
Male (*n* = 43)	−0.256	0.097	age	−0.099	0.532
Female (*n* = 12)	0.109	0.736	age	0.069	0.841
**TD-IQ**					
Total (*n* = 110)	−0.154	0.108	sex + age	−0.123	0.204
Male (*n* = 86)	−0.049	0.656	age	−0.032	0.772
Female (*n* = 24)	−0.312	0.078	age	−0.323	0.106

*Spearman partial correlation: adjusted for confounding factors in the table. ASD, autism spectrum disorders; IQ, intelligent quotient; n, number; OT, oxytocin; TD, typically developing. The bold value means significant p*.

### Oxytocin receptor (*OXTR*) gene rs2254298 polymorphism

The distribution of the *OXTR* SNP rs2254298 genotypes was consistent with the Hardy-Weinberg equilibrium for both groups (χ^2^ = 0.007, *df* = 2, *p* = 0.996 in ASD subjects; χ^2^ = 0.292, *df* = 2, *p* = 0.864 in TD controls). No statistical differences were found from the genotypic and allelic frequencies of the *OXTR* SNP rs2254298 in ASD subjects and TD controls (*p* > 0.05) (Table 3).

**Table 3:** **Participant genotypic and allelic frequencies of the ***OXTR*** SNP rs2254298 by group**.

**Group**	**Genotype**			**Allele**	
	**AA**	**AG**	**GG**	**A**	**G**
ASD subjects	14 (14.0%)	42 (42.0%)	44 (44.0%)	70 (35.0%)	130 (65.0%)
TD controls	24 (10.3%)	100 (43.1%)	108 (46.6%)	148 (31.9%)	316 (68.1%)
χ^2^	0.935			0.610	
*df*	2			1	
*p*	0.627			0.435	

*ASD, autism spectrum disorders; TD, typically developing*.

### Serum OT levels and *OXTR* SNP rs2254298 genotypes

**Figure 2:**
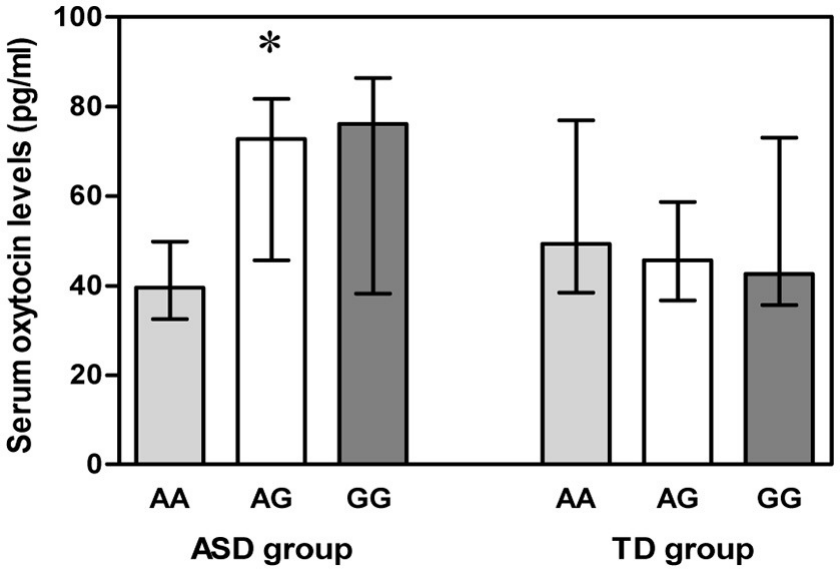
**Vertical bar charts of serum oxytocin levels for the three ***OXTR*** SNP rs2254298 genotypes in both ASD and TD groups are presented as median (IQR)**. Error bars show interquartile range (IQR). ASD group: *n* = 9 AA, *n* = 21 AG, and *n* = 25 GG. TD group: *n* = 9 AA, *n* = 52 AG, and *n* = 49 GG. The ASD subjects exhibited a significant association between serum oxytocin levels and three *OXTR* SNP rs2254298 genotypes, and serum oxytocin levels were low in ASD subjects with an AA genotype. ^*^p < 0.05. *ASD, autism spectrum disorders; IQR, interquartile range; OXTR, oxytocin receptor; SNP, single-nucleotide polymorphism; TD, typically developing*.

Both blood and DNA samples were available for 55 ASD subjects and 110 TD controls. A Kruskal-Wallis *H* test detected differences among the three genotypes in the ASD group (χ^2^ = 6.012, *df* = 2, *p* = 0.049) (Figure 2), but a Mann-Whitney U test with Bonferroni correction identified no evidence for significant associations between AA and AG genotypes (*p* = 0.080), AA and GG genotypes (*p* = 0.095), as well as AG and GG genotypes (*p* = 1.000).

### Correlations between serum OT levels and ASD social phenotypes

ABC scores and CARS scores were only determined for ASD subjects. Spearman correlation and Spearman partial correlation (adjusted for sex, age, and IQ) were both applied for all scores of the ABC and CARS. We found a significant positive correlation between serum OT levels and CARS total scores (Spearman correlation: *r* = 0.488, *p* = 0.000; Spearman partial correlation: *r* = 0.465, *p* = 0.001), suggestive of more social deficits with high serum OT levels. Moreover, serum OT levels also correlated positively with the “adaptation to change score” in the CARS (Spearman correlation: *r* = 0.352, *p* = 0.008; Spearman partial correlation: *r* = 0.296, *p* = 0.033), indicating that ASD subjects with higher serum OT levels were accompanied by worse abilities in adapting to novel environment. There were no significant corrections between serum OT levels and scores of other items in the CARS and ABC.

### *OXTR* SNP rs2254298 genotypes and ASD social phenotypes

**Figure 3:**
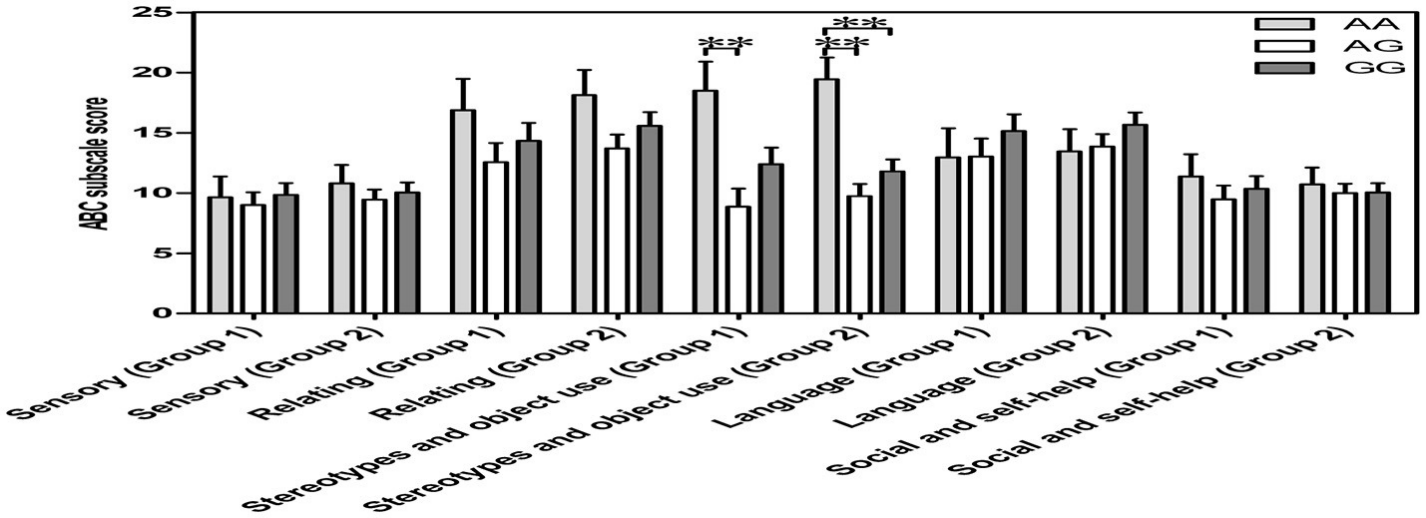
**Vertical bar charts of ABC subscale score among the three ***OXTR*** SNP rs2254298 genotypes are presented as mean ± SEM (adjusted for sex, age and IQ) in two groups**. Error bars show standard error of the mean (SEM). Group1 (ASD subjects for both OXT and *OXTR*): *n* = 9 AA, *n* = 21 AG, and *n* = 25 GG. Group 2 (ASD subjects for *OXTR*): *n* = 14 AA, *n* = 42 AG, and *n* = 44 GG. ASD subjects with an AA genotype exhibited a higher score than those with an AG genotype or a GG genotype. ^*^p < 0.05. ^**^p < 0.01. *ABC, Autism Behavior Checklist; ASD, autism spectrum disorders; OXT, oxytocin; OXTR, oxytocin receptor; SEM, standard error of the mean; SNP, single-nucleotide polymorphism*.

All scores for the 5 ABC categories conformed to a normal distribution and homogeneity of variance (*p* > 0.05) in two groups, including “ASD subjects for both OT and *OXTR*” (*n* = 55) and “ASD subjects for *OXTR*” (*n* = 100). In both groups, a one-way covariance (ANCOVA) with sex, age, and IQ as covariates indicated a significant relationship between genotypes and the “stereotypes and object use score” of the ABC [*F*_(2, 52)_ = 5.178, *p* = 0.006, *n* = 55; *F*_(2, 97)_ = 11.022, *p* = 0.000, *n* = 100]. Further the Bonferroni *post-hoc* test revealed significant differences between the AA and AG genotypes in both ASD groups with different sample sizes (“ASD subjects for both OT and *OXTR*” group: *p* = 0.005, *n* = 30; “ASD subjects for *OXTR*” group: *p* = 0.000, *n* = 56) as well as between the AA and GG genotypes in “ASD subjects for *OXTR*” group (*p* = 0.001, *n* = 58) (Figure 3). The score of subjects with an AA genotype was higher than that of subjects with AG or GG genotypes, indicating that the participants with an AA genotype exhibited worse behavioral deficits in stereotyped behavior and impaired ability of using objects.

A Kruskal-Wallis H test followed by Bonferroni-corrected Mann-Whitney U test was applied to make the comparisons of CARS total scores among three genotypes of *OXTR* SNP rs2254298, but no differences were found (*p* > 0.05). Moreover, a ordinal polytomous logistic regression analysis with *post-hoc* Bonferroni correction was used to analyze differences of scores of each item in the CARS among *OXTR* SNP rs2254298 genotypes (while adjusting for sex, age, and IQ). Significant associations of “adaptation to change score” in the CARS among three genotypes were found in ASD subjects. ORs (95% CI) for “4 = severely abnormal” of “adaptation to change” among three *OXTR* SNP rs2254298 genotypes in “ASD subjects for both OT and *OXTR*” (*n* = 55) and “ASD subjects for *OXTR*” (*n* = 100) groups were shown in Table 4. ASD subjects with an AA genotype acted as a protective genotype for suffering from the most severe abnormality of “adaptation to change,” indicating that ASD subjects with AG and GG genotypes tended to perform worse social cognition in novel environment.

**Table 4:** **Odds ratios (ORs) with 95% confidence intervals (CI) of having severe abnormality of 15 items in the CARS among three ***OXTR*** SNP rs2254298 genotypes in two ASD groups**.

**Items of CARS**	**Ref**	**ASD subjects for both OT and *OXTR* (*n* = 55)**			**ASD subjects for *OXTR* (*n* = 100)**		
		**AA vs. AG**	**AA vs. GG**	**AG vs. GG**	**AA vs. AG**	**AA vs. GG**	**AG vs. GG**
Relating to people	4	2.34 (0.34, 16.02)	1.33 (0.41, 4.30)	0.57 (0.22, 1.47)	0.75 (0.19, 3.04)	0.73 (0.30, 1.77)	0.97 (0.51, 1.84)
Imitative behavior	4	0.91 (0.16, 5.31)	0.91 (0.30, 2.72)	0.99 (0.43, 2.29)	0.47 (0.12, 1.84)	0.63 (0.27, 1.49)	1.33 (0.72, 2.44)
Emotional response	4	0.29 (0.03, 2.81)	0.39 (0.09, 1.66)	1.37 (0.49, 3.79)	0.70 (0.19, 2.64)	0.60 (0.26, 1.39)	0.85 (0.46, 1.57)
Body use	4	1.15 (0.19, 6.88)	1.09 (0.36, 3.32)	0.95 (0.41, 2.20)	1.60 (0.45, 5.72)	1.13 (0.51, 2.52)	0.71 (0.39, 1.28)
Object use	4	0.84 (0.13, 5.49)	0.83 (0.26, 2.69)	0.99 (0.41, 2.38)	0.27 (0.07, 1.07)	0.60 (0.15, 1.06)	0.71 (0.39, 1.28)
Adaptation to change	4	0.07 (0.01, 0.50)^**^	0.22 (0.07, 0.77)^*^	3.24 (1.36, 7.69)^**^	0.12 (0.03, 0.45)^**^	0.30 (0.13, 0.71)^**^	2.60 (1.43, 4.73)^**^
Visual response	4	1.03 (0.17, 6.37)	1.09 (0.35, 3.41)	1.05 (0.44, 2.50)	1.70 (0.48, 6.01)	1.29 (0.58, 2.85)	0.761 (0.42, 1.37)
Listening response	4	1.46 (0.29, 7.39)	1.17 (0.43, 3.20)	0.80 (0.37, 1.72)	2.45 (0.74, 8.05)	1.76 (0.83, 3.71)	0.72 (0.42, 1.24)
Perceptive response	4	1.04 (0.19, 5.64)	0.72 (0.25, 2.06)	0.69 (0.31, 1.55)	0.92 (0.28, 3.02)	0.79 (0.38, 1.68)	0.86 (0.50, 1.50)
Fear or anxiety	4	0.60 (0.11, 3.30)	0.76 (0.26, 2.17)	1.26 (0.56, 2.84)	0.43 (0.13, 1.47)	0.58 (0.27, 1.26)	1.34 (0.76, 2.34)
Verbal communication	4	0.43 (0.07, 2.75)	0.61 (0.19, 1.91)	1.40 (0.59, 3.33)	0.43 (0.11, 1.63)	0.55 (0.24, 1.27)	1.27 (0.69, 2.32)
Non-verbal communication	4	0.60 (0.10, 3.48)	0.70 (0.24, 2.10)	1.18 (0.51, 2.69)	0.90 (0.25, 3.23)	0.96 (0.43, 2.14)	1.07 (0.59, 1.92)
Activity level	4	0 (0, 3.24E+160)	0 (0, 8.43E+106)	29.37 (0, 3.32E+56)	0.16 (0.02, 1.62)	0.26 (0.06, 1.18)	1.63 (0.66, 4.02)
Level and consistency of intellective relation	4	0.72 (0, 1.25E+36)	0.91 (0, 1.54E+24)	1.27 (0, 2.59E+12)	5.36 (0, 1.34E+43)	3.37 (0, 6.04E+28)	0.63 (0, 9.46E+13)
General impressions	4	2.44 (0.30, 19.59)	1.66 (0.46, 6.00)	0.68 (0.25, 1.82)	1.75 (0.39, 7.88)	1.21 (0.47, 3.11)	0.69 (0.35, 1.38)

*Rating of 15 items in CARS with a four-point scale (1 = appropriate for age; 2 = mildly abnormal; 3 = moderately abnormal; 4 = severely abnormal). The reference (Ref) rating is 4 in a ordinal polytomous logistic regression analysis. ASD subjects for both OXT and OXTR: n = 9 AA, n = 21 AG, and n = 25 GG. ASD subjects for OXTR: n = 14 AA, n = 42 AG, and n = 44 GG*.

* p < 0.05;

** *p < 0.01. ASD, autism spectrum disorders; CARS, Childhood Autism Rating Scale; n, number; OT, oxytocin; OXTR, oxytocin receptor; Ref, reference; vs, versus*.

## Discussion

Animal and human studies, including studies of individuals with ASD, have provided evidence for the essential role of OT and the *OXTR* gene in social functioning. In the present study, we tested serum OT levels of both genders and elevated serum OT levels were observed in the ASD group compared to the TD group. Serum OT levels correlated positively with the “adaptation to change score” and “total scores” of the CARS. No associations were found between serum OT levels and age, as well as IQ in both ASD and TD groups. In addition, we made a comparison of the *OXTR* rs2254298 polymorphism in both groups and observed no statistical significance. However, significant associations were detected between *OXTR* SNP rs2254298 genotypes and serum OT levels, the “stereotypes and object use score” of the ABC and the “adaptation to change score” of the CARS in the ASD subjects.

Our finding of higher serum OT levels in ASD participants was similar to several previous studies on plasma OT levels in individuals with ASD. For example, Jansen et al. (2006) observed increased plasma OT levels in a group of male and female ASD adults, and explained that developmental, intellectual, or other factors as well as a physiological reaction on negative moods and stress may relate to the higher OT plasma levels in ASD participants. Thus far, evidence has suggested elevated peripheral OT levels in humans and animals in connection with depression, anxiety and social stress (Grippo et al., 2007; Holt-Lunstad et al., 2011; Miller et al., 2013; Weisman et al., 2013) as well as a relationship between OT levels and altered energetic stress coping approaches and anxiolytic influences (Insel, 2010; Neumann and Landgraf, 2012; Lukas and Neumann, 2013). Exposure to unfamiliar environments is well-known to be stressful for individuals with ASD, which can trigger a stress response to release central OT to counter negative emotions (Gimpl and Fahrenholz, 2001). We could not find the correlations between serum OT levels and age in the ASD group (including males and females) after adjusting for IQ, which predicted that the intellectual factors might be restrictive factors for serum OT levels and developmental stage. However, we detected higher serum OT levels were associated with worse ability in adapting to change and greater social deficits (as evidenced by high CARS total scores) in ASD subjects, predicting impairments in social functioning may lead them to release more OT to withstand different novel environments. Furthermore, Jacobson et al. (2014) reported that prepubertal male children with ASD exhibit elevated plasma OT levels and increased G protein subunit expression relative to TD controls and higher oxytocin levels associated with greater social impairment. We conjectured that a dysregulation in OT signaling pathways likely including a potential compensatory mechanism for the changes of *OXTR* gene expression may also lead to elevated OT levels in ASD subjects. That is why we designed experiments on both serum OT levels and an *OXTR* gene polymorphism to find latent disturbances in ASD individuals.

However, Miller et al. (2013) reported that girls exhibit higher plasma OT levels and elevated anxiety scores than boys within an autistic group instead of the difference between the cases and controls. Similarly, Taurines et al. (2014) also did not observe differences in plasma OT levels of children and adolescents between high-functioning male ASD subjects and healthy male controls, nor did Parker et al. (2014) among ASD participants, unaffected siblings and unrelated neurotypical control children of both genders. Moreover, four studies have exhibited lower plasma OT levels. Modahl et al. (1998) reported lower plasma OT levels in prepubertal boys with ASD relative to TD boys, but ASD participants with the highest OT levels were the most socially impaired. Moreover, they found a positive association between OT levels and age for normal children, which was opposite from our finding. Green et al. (2001) observed a decrease in OT and an increase in unprocessed OT peptides in male autistic children, relative to controls. Andari et al. (2010) elucidated lower plasma OT levels in young adults with high-functioning autism (HFA) or Asperger Syndrome, and Al-Ayadhi (2005) reported lower plasma levels of OT in children and adolescents with ASD.

Overall, a substantial body of research has reported inconsistent outcomes in peripheral OT levels, which could be the result of differences among participants and investigative methods, including the multiple demographic characteristics of the ASD population (age, gender, intelligence, ethnicity, comorbidity, medication history, sample size, diagnostic criteria, etc.), the blood sample collection time and the measurement and analysis of OT levels. Decreased OT levels in ASD might be a manifestation of the original genetic insult of ASD, and elevated OT levels in ASD might reflect a physiological response to ameliorate negative symptoms such as anxiety (Taurines et al., 2014) and be consistent with some degree of a dysregulation in OT peptide processing pathways (Green et al., 2001). Because plasma OT concentrations are highly heritable (Parker et al., 2014), family effects should be taken into consideration in subsequent studies.

With the aim of establishing the role of the *OXTR* gene in ASD, we chose to study a particularly promising candidate: *OXTR* SNP rs2254298. Many studies have reported that *OXTR* SNP rs2254298 affects various psychological dimensions and neurological diseases, such as affect, temperament, genetic variation, attachment and social cognition. However, there is extant literature failing to associate the *OXTR* rs2254298 polymorphism with ASD consistently.

The first association study examining 4 SNPs in 195 Chinese Han autism trios with a family-based association test (FBAT) revealed a significant genetic association with rs2254298A and rs53576A (Wu et al., 2005). A Japanese study only observed a significant difference in rs2254298A between the ASD and normal groups in a population-based case-control test but not in an FBAT (Liu et al., 2010). A study of 57 American Caucasians attempted to replicate the Asian studies but only detected an association with rs2254298G (Jacob et al., 2007). Four studies did not identify any direct associations between the *OXTR* rs2254298 polymorphism and ASD. A comprehensive study of 18 tagged SNPs across the entire *OXTR* gene region in 152 Israeli autistic subjects was undertaken using HapMap data, the Haploview algorithm, a case-control test, and a FBAT. A five-locus haplotype block (rs237897-rs13316193-rs237889-rs2254298-rs2268494) was significantly associated with ASD, but SNP rs2254298 in the case-control test did not reveal any association with ASD except for the relationship with “daily living skills” and “communication” scores of the Vineland Adaptive Behavior Scales (Lerer et al., 2008). A study in northern European Caucasians reported the association of Asperger Syndrome with multiple haplotypes that included rs2254298, but no association between rs2254298 alone with Asperger Syndrome was observed (Di Napoli et al., 2014). Another study on HFA in Swiss Caucasians between 5 and 17 years of age confirmed no association between rs2254298 and ASD (Nyffeler et al., 2014); this finding was similar to the finding in a family-based single-marker and haplotype association study with 22 SNPs in 100 German families with a high-functioning autistic proband (Wermter et al., 2010).

Our study did not detect any association between rs2254298 and ASD, which was similar to the conclusion of a meta-analysis: a solid role in autism has not been established for *OXTR* rs2254298 (Bakermans-Kranenburg and van Ijzendoorn, 2014). However, after the analyses of the genotypes among serum OT levels and the scores for ABC and CARS, some interesting associations were detected in the present study. A significant association between serum OT levels and *OXTR* rs2254298 polymorphism was observed in the ASD group, and the serum OT levels of an AA genotype showed the potential to be lower than the AG and GG genotypes, which suggested that a dysfunction of *OXTR* SNP rs2254298 in ASD subjects may somewhat impact OT levels in *OXTR*-OT signaling pathways. The score for “stereotypes and object use” in the ABC revealed significant differences among the three genotypes in two groups with different sample sizes. The severity of behavioral deficits in ASD participants with an AA genotype was significantly greater than the AG genotype in “ASD subjects for both OT and *OXTR*” (*n* = 55) group, and the severity of behavioral deficits in ASD participants with an AA genotype was significantly greater than the AG and GG genotypes in “ASD subjects for *OXTR*” (*n* = 100) group. Although no evidence for a direct association between serum OT levels and “stereotypes and object use score,” we found ASD subjects with AG and GG genotypes tend to have higher OT levels and lesser behavioral impairments (including restricted and repetitive behaviors). These results appeared to be consistent with the reduced repetitive behaviors after intranasal OT infusion (Hollander et al., 2003). Furthermore, the score of “adaptation to change” in the CARS was also found to be statistically significant in two groups with different sample sizes, and ASD subjects with an AA genotype were better able to adapt to change than those with AG and GG genotypes. Along with a significant correction between serum OT levels and “adaptation to change score,” we also found ASD subjects with an AA genotype tend to show lower OT levels and lesser social cognitive impairment compared to those with AG and GG genotypes. This result might stem from the different reactions to different novel environments along with different severity of social cognitive deficits (Tops et al., 2013). Above all, we found the ASD subjects with an AA genotype have the better ability to adapt to change, as a result, they might produce less OT to deal with different novel environments. However, elevated OT levels can reduce repetitive behaviors in ASD patients (Hollander et al., 2003), and this may be the reason for higher score of “stereotypes and object use” in ASD subjects with an AA genotype. On the whole, we speculate that differences in serum OT levels and social deficits among *OXTR* SNP rs2254298 genotypes likely involve changes at the *OXTR* expression as well as environmentally induced alterations of the basal oxytocinergic system in ASD.

The first strength of our study lies in the combination of the *OXTR* rs2254298 polymorphism with serum OT levels in both ASD and TD groups as well as the effect of the *OXTR* SNP rs2254298 on ASD social phenotypes. Our results could to some extent explain the potential role of OT in the impairment of social and behavioral skills in ASD (Bartz and Hollander, 2008) and provide ideas to explain the opposite emotion recognition performance after OT administration between different *OXTR* haplotypes (Chen et al., 2015). Secondly, both age- and gender-matched males and females participated in the study, which extended the findings of most previous studies that involved only males. Thirdly, a standardized protocol for blood and DNA collection with fasting in the morning was used to preclude potential circadian and contaminating effects on serum OT levels and DNA quality.

There are three limitations of our study. First, we chose serum rather than CSF as the test sample, and serum OT levels can not completely reflect OT actions in the central nervous system because the relationship between peripheral and central levels of OT is unclear. Second, we only got informed consent from 55 ASD subjects and 110 TD controls to measure both serum OT levels and the *OXTR* rs2254298 polymorphism, which needs to expand the sample size in further studies. Finally, the PLINK program was not performed in our study, we had to calculate the allele frequencies by hand and then do case-control association analysis by a χ^2^-test using SPSS. Our findings will need to be replicated in East Asian samples and very large samples will be required to examine these associations in European samples.

In summary, to the best of our knowledge, our study is the first to test serum OT levels in ASD participants and analyze the association between the *OXTR* rs2254298 polymorphism and serum OT levels, the ABC scores and the CARS scores. ASD subjects exhibited elevated serum OT levels and a latent association among OT levels, the *OXTR* rs2254298 polymorphism and ASD social deficits existed in ASD group. Although we did not detect any direct association between the *OXTR* rs2254298 polymorphism and ASD, between serum OT levels and age, as well as and between serum OT levels and IQ, we could indentify some clues to indicate social deficits in ASD individuals with OT levels and the *OXTR* rs2254298 polymorphism. Subsequent prospective studies should focus on larger, well-described populations of different races to elucidate the true effects of the OT and *OXTR* gene on ASD under the considerations of genetic, environmental and developmental factors, then design a reasonable therapeutic schedule for the oxytocinergic system to improve their social deficits.

## Author contributions

SY contributed to the sample collection, laboratory experiments, acquisition of data, analysis and interpretation of data, and preparation of the first draft of manuscript. XD, XG, and YH contributed to sample collection, laboratory experiments, acquisition of data, analysis and interpretation of data. HS and LG contributed to acquisition of data, analysis and interpretation of data, and revision of the manuscript for important intellectual content. WD and YS contributed to the conception and design of the study, sample collection, analysis and interpretation of data, and revision of the manuscript for important intellectual content. XZ contributed to the conception and design of the study, sample collection, acquisition of data, analysis and interpretation of data, and review and revision of the manuscript. All authors read and approved the final manuscript.

## Funding

This research was supported by grants from the National Natural Science Foundation of China (No.81072313).

### Conflict of interest statement

The authors declare that the research was conducted in the absence of any commercial or financial relationships that could be construed as a potential conflict of interest.
